# Pathogen-associated heterogeneity in pulmonary sepsis: from inflammatory phenotypes and transcriptomic endotypes to precision medicine

**DOI:** 10.3389/fimmu.2026.1973456

**Published:** 2026-10-16

**Authors:** Chun Wang, Cheng Xi, Ying Zhang, Ruifang Ma, Mimi Shi, Yu Gao, Liping Liu, Canyu Ning, Anbao Chen

**Affiliations:** 1 Department of Emergency Intensive Care Unit, Second Affiliated Hospital of Kunming Medical University, Kunming, Yunnan, China; 2 Department of Gastrointestinal Surgery, First Affiliated Hospital of Kunming Medical University, Kunming, Yunnan, China; 3 Department of Emergency, Second Affiliated Hospital of Kunming Medical University, Kunming, Yunnan, China

**Keywords:** endotype, inflammatory phenotype, pathogens, precision medicine, pulmonary sepsis

## Abstract

Sepsis is a heterogeneous syndrome, and pulmonary infection is its commonest source. Pulmonary sepsis carries a particularly high mortality, especially in patients who develop refractory septic shock, yet it is still managed as a single disease. Conventional diagnostic criteria, based on organ dysfunction, describe the consequences of the host response rather than its biological diversity, and this mismatch helps to explain why immunomodulatory trials have repeatedly failed in unselected patients. In this review we summarise how pathogen characteristics interact with the alveolar immune microenvironment to produce distinct host-response phenotypes, and how those phenotypes are captured by clinical classifiers, plasma protein biomarkers and blood transcriptomic endotypes — among them SRS1/2, Mars1–4, SENECA and USE-1/2. Three problems currently limit translation. The classification systems overlap only in part, and should not be used interchangeably. Endotypes change over time, so a single measurement at baseline can misclassify a patient. And most of the evidence linking particular pathogens to particular phenotypes is associative, and comes from ARDS, general sepsis, severe pneumonia or COVID-19 cohorts rather than from pulmonary sepsis itself. We then review the phenotype-dependent responses to fluid strategy, corticosteroids, statins, endotoxin removal, cytokine-directed therapy and checkpoint inhibition, and consider why several biomarker-guided trials produced pharmacodynamic signals without clinical benefit. Pathogen-informed, repeatedly measured host-response phenotypes, tested in adaptive trials with enrichment, offer the most realistic route to precision immunotherapy in pulmonary sepsis.

## 1 Introduction

Sepsis remains a formidable critical care challenge, with an estimated 48.9 million incident cases and 11.0 million sepsis-related deaths each year, accounting for 19.7% of all deaths globally (1, 2). The clinical manifestations, disease course, and prognosis of sepsis are closely related to the source of infection. Although infections originating from different anatomical sites—such as the lungs, abdomen, urinary tract, and central nervous system—can all trigger systemic inflammatory response syndrome, there are significant differences in host immune response patterns, the spectrum of organ damage, and clinical outcomes (3). However, among all sources of infection leading to sepsis, pulmonary infections are the most common, accounting for approximately 60% of cases (3, 4). The pathological process in which a localized respiratory tract infection spreads systemically is referred to as pulmonary sepsis, which is also a major cause of acute respiratory distress syndrome (ARDS) and multiple organ failure.

In the past, the diagnosis and treatment of pulmonary sepsis primarily treated the condition as a homogeneous process of uncontrolled inflammation (5, 6). However, the successive failures of clinical trials over the past few decades targeting lipopolysaccharide, tumor necrosis factor-α (TNF-α), and other molecules have prompted the academic community to deeply reflect on the fact that, while diagnostic criteria based on organ dysfunction scores are universally applicable, they obscure the underlying pathophysiological heterogeneity of the disease (5, 7). Consensus methodological recommendations for sepsis phenotype discovery have recently been published, emphasizing the need for standardized latent class analysis and external validation across diverse cohorts to enhance reproducibility and clinical translation (8).

Based on an in-depth understanding of the heterogeneity of sepsis, multi-omics and machine learning clustering algorithms have led to the emergence of the concepts of “phenotype” and “endotype.” A phenotype refers to a subgroup of patients with similar clinical trajectories, classified based on observable characteristics such as clinical variables and biomarkers (9). An endotype, in turn, delves further into the molecular biological mechanisms and pathways that drive disease progression. In recent years, groundbreaking research has revealed that the host’s inflammatory phenotype is not determined solely by genetic susceptibility and underlying diseases; specific characteristics of the pathogen—such as species, viral load, virulence factors, and site of colonization—play a crucial and decisive role in shaping the phenotype (10). Phenotypic classification that ignores microbiological data is inherently incomplete. Integrating pathogen characteristics into host response models not only improves the homogeneity of phenotypic clustering but also enables personalized precision medicine for sepsis.

Although emerging frameworks emphasize multi-omic integration to advance sepsis precision medicine (11), critical gaps remain regarding how specific pathogen factors drive distinct pulmonary phenotypes. This review provides a focused synthesis of the mechanisms underlying inflammatory phenotypes and transcriptomic endotypes in pulmonary sepsis, and discusses their translational potential for individualized risk stratification and targeted therapy. The pathogen-associated phenotypes, the classification systems used to describe them and the corresponding therapeutic options are summarized in **Tables 1**–**3**. In this review we use the term pathogen-associated heterogeneity to denote a framework in which pathogen identity, microbial load and virulence factors are treated as determinants of the host response, while noting that most of the supporting evidence remains associative rather than causal.

**Table 1:** Pathogen-associated host inflammatory phenotypes and the evidence linking each pathogen class to them.

Pathogen class	Dominant PAMPs → PRRs	Immunopathological mechanism	Phenotype/endotype evidence	Population studied	Evidence level	References
Gram-negative Bacteria(e.g., E. coli, K. pneumoniae)	LPS (Lipid A)→ TLR4/MD2/CD14	OMV-mediated LPS delivery; explosive NF-κB activation; surge of TNF-α, IL-1β, IL-6, and tissue factors	Highest probability of the hyperinflammatory phenotype (median probability 0.50 for E. coli vs 0.14 for S. aureus); endotoxin activity correlates with inflammatory intensity	Pulmonary and bloodstream infection; mixed sepsis cohorts	Direct for phenotype; no transcriptomic endotype distribution reported	(10, 44, 45, 47, 64, 71, 73)
Gram-positive Bacteria(e.g., S. aureus, S. pneumoniae)	PGN, LTA, Exotoxins→ TLR2/TLR1 or TLR6	Exotoxin/Pneumolysin-induced alveolar epithelial pyroptosis via GSDMD/NLRP3 inflammasome	Lower probability of the hyperinflammatory phenotype; higher S100A12 and platelet counts; pneumolysin-driven alveolar epithelial pyroptosis in experimental models	Bloodstream infection; experimental pneumonia models	Direct for phenotype; experimental for mechanism; no direct endotype data	(10, 44, 46, 48)
Respiratory Viruses(e.g., SARS-CoV-2, Influenza)	Viral RNA/DNA→ RIG-I, MDA5, TLR3/7/8	Type I IFN surge; dysregulated CD8+ T cell differentiation; CD38/CD73-driven adenosine immunosuppression	Type I interferon surge; dysregulated CD8+ T-cell differentiation; CD38/CD73-driven adenosine-mediated immunosuppression; severe lymphopenia	COVID-19 and viral sepsis cohorts; not pulmonary sepsis specifically	Direct for viral sepsis; indirect for pulmonary sepsis	(49–53, 66–68, 75)
Invasive Fungi(e.g., Candida spp., Aspergillus spp.)	β-glucans, Mannans→ Dectin-1, CLRs	Upregulation of PD-1/CTLA-4 immune checkpoints; Wnt/ROS-induced APC exhaustion; T/B-cell apoptosis	Upregulation of PD-1/PD-L1 and CTLA-4; loss of HLA class II and of CD28/CD27; Wnt/ROS-associated APC dysfunction — a phenotype resembling the immunosuppressed SRS1 endotype	Mixed ICU cohorts with secondary fungal infection; sepsis-induced immunosuppression	Indirect: no direct evidence in pulmonary sepsis; no transcriptomic endotype distribution reported	(54–60, 76)

**Table 2:** Comparison of the principal sepsis classification systems.

Classification	Derivation cohort	Data layer	Key features	Prognostic direction	Therapeutic implication	Cross-validation with other systems
SRS1/SRS2	GAinS (pneumonia and peritonitis); 265 discovery, 106 validation	Whole-blood transcriptome	SRS1: endotoxin tolerance, T-cell exhaustion, HLA class II downregulation; SRS2: relatively preserved adaptive immunity	SRS1: higher 14-day mortality (HR 2.4–2.8)	VANISH: harm with hydrocortisone in SRS2 (OR 7.9); SRS1 proposed as a target for immunostimulation	Partially compared with the hyper-/hypoinflammatory phenotypes; not equivalent
Mars1–4	MARS (Netherlands); validated in a Brazilian multicenter cohort	Whole-blood transcriptome	Mars1: immunometabolic dysregulation	Mars1: 28-day mortality up to 39–52.8%; validation cohort HR 1.78	Enrichment for immunostimulation proposed; not tested prospectively	Not systematically compared with SRS or clinical phenotypes
SENECA (α, β, γ, δ)	US electronic health record cohort (~60,000 patients)	Clinical variables and routine laboratory data	δ subtype: refractory hypotension, hyperlactatemia, hepatic and cardiovascular failure	δ subtype: highest mortality; α and β mildest	Simulation-based treatment implications only	δ subtype broadly overlaps the hyperinflammatory phenotype; not interchangeable
Hyper-/hypoinflammatory	ARDS cohorts (HARP-2, FACTT, ALVEOLI, VALID)	Clinical variables plus plasma protein biomarkers	Hyperinflammatory: high IL-6, IL-8 and sTNFR1, low bicarbonate; hypoinflammatory: lower inflammation, higher MMP-8	Hyperinflammatory: higher mortality in ARDS cohorts	Differential response to fluid strategy, simvastatin and PEEP reported	Partial overlap with SRS endotypes; prognostic directions may diverge
USE-1/USE-2	Two Ugandan cohorts	Whole-blood transcriptome	Two transcriptomic endotypes with global alignment attempted	One endotype associated with higher mortality	Not tested	Alignment with SRS and Mars attempted, not established
Coagulopathic/adaptive/inflammatory	Multi-cohort re-analysis of bacterial sepsis transcriptomes	Whole-blood transcriptome	Coagulopathic, adaptive (T-cell rich) and inflammatory clusters	Coagulopathic cluster: highest mortality (30% vs 16%)	Not tested prospectively	Not cross-validated with the clinical frameworks

**Table 3:** Treatment-by-endotype matrix for pulmonary sepsis.

Intervention	Target phenotype/endotype	Level of evidence	Key trial(s) and result	Situations in which it should not be used
Conservative fluid strategy	Hypoinflammatory phenotype	Secondary analysis of a randomized trial	FACTT: 90-day mortality 18% (conservative) vs 26% (liberal) in the hypoinflammatory subphenotype; 50% vs 40% in the hyperinflammatory subphenotype (p = 0.0039 for interaction)	Should not be applied uniformly: in the hyperinflammatory subphenotype the conservative strategy was associated with higher mortality
Corticosteroids (hydrocortisone)	SRS2/immunocompetent; immunoadaptive (IA-P)	Secondary analyses of randomized trials; one phase 3 trial in severe CAP	VANISH: increased mortality with hydrocortisone in SRS2 (OR 7.9); ADRENAL secondary analysis: increased mortality in the pulmonary-sepsis subgroup with the IA-P phenotype (OR 5.55, 95% CrI 1.81–21.2); CAPE COD: reduced 28-day mortality in severe community-acquired pneumonia (6.2% vs 11.9%)	Evidence for phenotype-directed use is not established: a subgroup analysis suggested harm in the immunoadaptive phenotype, while the overall analysis found no heterogeneity of treatment effect
Simvastatin	Hyperinflammatory phenotype (ARDS)	Secondary analysis of a randomized trial (primary trial negative)	HARP-2 secondary analysis: higher 28-day survival with simvastatin in the hyperinflammatory subphenotype; no difference in the hypoinflammatory subphenotype	Not indicated outside a trial in the hypoinflammatory phenotype
Polymyxin B hemoperfusion (PMX-HA)	High endotoxin activity (EAA 0.60–0.89)	Exploratory *post hoc* analysis of a randomized trial (primary trial negative)	EUPHRATES *post hoc*: lower adjusted 28-day mortality with PMX-HA in patients with EAA 0.60–0.89	Should not be selected on the basis of inflammatory phenotype; requires prospective confirmation
IL-6 receptor antagonists (tocilizumab)	Viral hyperinflammatory states	Randomized trial in COVID-19	RECOVERY: reduced mortality in hypoxic COVID-19 patients receiving corticosteroids	No evidence of benefit in bacterial pulmonary sepsis; should not be extrapolated
Anti-PD-1/anti-PD-L1 checkpoint inhibition	SRS1 (immunosuppressed endotype)	Phase 1b safety and pharmacodynamic study	Single ascending dose anti-PD-L1 study: acceptable short-term safety, preliminary pharmacodynamic signals	Not for routine use; clinical benefit unproven; should not be given without endotype enrichment
Recombinant IL-7	SRS1/lymphopenic immunosuppression	Phase 2 pharmacodynamic evidence; clinical benefit not established	Improvement in lymphocyte counts without consistent clinical benefit	Not for unselected patients; timing of administration not established
GM-CSF and thymosin α1	Fungal pulmonary sepsis (proposed)	Limited and conflicting evidence; no trial in pulmonary sepsis	Small studies in selected populations; reports of both benefit and harm for GM-CSF	Should not be presented as established precision therapies; evidence insufficient to recommend use outside trials

*: Host responses are dynamic; clinical phenotypes and transcriptomic endotypes may transition over time (e.g., endotype drift from SRS1 to SRS2). Therapeutic strategies should be guided by longitudinal monitoring of patient status rather than baseline evaluation alone (40, 42).

This table outlines the distinct pathophysiological trajectories initiated by four major pathogen classes in pulmonary sepsis. It maps the continuum from specific pathogen-associated molecular patterns (PAMPs) and their interactions with host pattern recognition receptors (PRRs), to the ensuing immune cascades and defining clinical phenotypes. Evidence-based biomarker profiles for risk stratification and corresponding targeted interventions are summarized to guide personalized immunomodulatory therapies and improve clinical outcomes. Abbreviations: AECs, alveolar epithelial cells; AMs, alveolar macrophages; APC, antigen-presenting cell; CAP, community-acquired pneumonia; CTLA-4, cytotoxic T-lymphocyte-associated protein 4; ECs, endothelial cells; GSDMD, gasdermin D; HLA-DR, human leukocyte antigen-DR isotype; IL, interleukin; LPS, lipopolysaccharide; MDA5, melanoma differentiation-associated protein 5; MVs, membrane vesicles; NETs, neutrophil extracellular traps; NF-κB, nuclear factor kappa B; NLRP3, NLR family pyrin domain containing 3; PD-1, programmed cell death protein 1; PD-L1, programmed death-ligand 1; PMX-HA, polymyxin B hemoperfusion; RIG-I, retinoic acid-inducible gene I; ROS, reactive oxygen species; SP-D, surfactant protein D; sRAGE, soluble receptor for advanced glycation end products; SRS1/2, sepsis response signature subtypes 1 and 2; sTNFR1, soluble tumor necrosis factor receptor-1; TLR, Toll-like receptor; TNF-α, tumor necrosis factor-alpha.

## 2 Clinical characteristics of pulmonary sepsis

### 2.1 Epidemiology and prognosis of pulmonary sepsis

#### 2.1.1 Epidemiological data identifying pulmonary infections as the primary cause

Pulmonary infections are the predominant trigger for sepsis.

In the international multicenter MARS cohort, sepsis of respiratory origin accounted for 53.8%, far outpacing abdominal (25.6%) and urinary tract (7.1%) sources (4). This predominant role is strongly corroborated by the Japanese FORECAST study involving 1,184 patients with severe sepsis and septic shock, where pulmonary infections ranked first at 31.0%, followed by abdominal (26.3%) and urinary tract (18.4%) infections (12). Furthermore, a large prospective study of 1,296 ICU patients similarly identified the lungs as the primary site of infection (13). This dominance persists across varied clinical contexts; even in surgical populations where intra-abdominal sepsis is inherently frequent, pulmonary infections remain a substantial driver at 19% (14). Regional data further corroborate this global pattern, with pulmonary sources comprising over 35% of sepsis admissions in independent cohorts (15).

This epidemiological dominance goes beyond mere statistics, representing a primary source of clinical heterogeneity in critical care. Because the respiratory tract interfaces directly with the environment, it is exposed to an extensive spectrum of bacterial, viral, and fungal pathogens, which in turn incite divergent host immune trajectories. Consequently, viewing pulmonary sepsis as a monolithic syndrome is fundamentally inadequate. A granular dissection of this population through pathogen-associated inflammatory phenotypes and transcriptomic endotypes is therefore essential, providing the conceptual framework for this review.

#### 2.1.2 Source-specific host responses highlighting the uniqueness of pulmonary sepsis

Although sepsis from different anatomical sites shares common systemic pathways, the host immune response is profoundly shaped by the primary focus of infection, with pulmonary sepsis exhibiting a highly distinct biological signature. Leukocyte transcriptome analysis from the prospective MARS cohort revealed that while pulmonary and abdominal infections share approximately 76% of their differentially expressed genes, the remaining 24% represent crucial, source-specific divergences in transcriptomic pathways governing hemostasis, innate/adaptive immunity, and cellular metabolism (4). Specifically, while abdominal sepsis triggers intense systemic inflammation and coagulation cascade cross-activation—driven by peritoneal exposure and bacterial translocation—pulmonary sepsis presents a more localized, tissue-specific pathological profile.

This distinct immunopathological trajectory is further supported by clinical kinetic data. In a prospective longitudinal study of 316 surgical sepsis patients, host response patterns varied dramatically by infection site. Compared to intra-abdominal infections, which are characterized by prolonged systemic pro-inflammatory states and protracted immunoparalysis, patients with pulmonary sepsis demonstrated a faster resolution of systemic immunosuppression and a quicker return to baseline systemic immunocompetence (14). Furthermore, while endothelial cell activation represents the predominant feature in urinary tract and skin infections, the systemic turmoil in pulmonary sepsis remains intimately tethered to localized respiratory damage.

In summary, whereas non-pulmonary sources (such as abdominal or urinary tract infections) primarily manifest as systemic endothelial injury and systemic inflammation-coagulation cross-activation (16), pulmonary sepsis is distinctively characterized by localized alveolar epithelial barrier disruption and the intensive remodeling of the respiratory immune microenvironment. This fundamental difference in host response localization underscores why immunomodulatory therapies designed for systemic inflammation frequently fail when applied uniformly to patients with pulmonary-origin sepsis.

#### 2.1.3 Lethal dynamics and shock-dependent mortality stratification in pulmonary sepsis

Beyond its high incidence, pulmonary sepsis poses a formidable clinical challenge due to its unique and aggressive mortality trajectory, which stands out drastically from non-pulmonary infections. Population-level evaluations from the US Multiple Cause of Death Database (2004–2018) underscores this immense public health burden, revealing that the age-adjusted mortality rate for pulmonary-origin sepsis reached a staggering 111.8 per million, capturing more than double the lethal toll of abdominal (46.7 per million) and genitourinary sepsis (52.0 per million) (16). When tracking acute clinical outcomes within intensive care environments, the eICU Collaborative Research Database confirms this persistent lethality, capturing an in-hospital mortality rate of 19.27% for pulmonary sepsis, which was higher than urinary tract sources (12.81%) and mirrors the high-risk nature of intra-abdominal catastrophe (18.93%) (17).

Importantly, granular risk stratification demonstrates that pulmonary sepsis exhibits a volatile, shock-dependent mortality dynamic. A landmark comparative analysis highlighted that in the *absence* of clinical shock, a pulmonary infection focus carries a disproportionately high and insidious risk of death compared to an abdominal focus (32.9% vs. 8.8%, *P* < 0.001). When patients deteriorate into refractory septic shock, the mortality rate for the pulmonary subgroup surges violently to 63.7% (vs. 48.4% for abdominal sepsis), establishing it as one of the most lethal phenotypic trajectories in critical care medicine (18).

This distinct lethal efficiency is fundamentally dictated by source-specific organ failure patterns. While non-pulmonary sources selectively trigger systemic pathways leading to widespread coagulopathy or acute kidney injury requiring renal replacement therapy (19), pulmonary sepsis converges directly and aggressively on refractory respiratory failure and ARDS. Consequently, the root cause of this severe clinical outcome stems from the high localized microbial load and specialized pathogen translocation pathways that cause structural collapse of the respiratory system before widespread systemic vascular shock fully manifests (20).

### 2.2 Differences in epithelial-endothelial damage in pulmonary sepsis

Differences in the site of origin fundamentally dictate the molecular characteristics of target organ damage. Based on the route of origin, sepsis can be broadly classified into pulmonary sepsis and non-pulmonary sepsis (triggered by abdominal, urinary, or bloodstream infections).

The pathogenesis of pulmonary sepsis is primarily characterized by direct damage to alveolar epithelial cells. In these patients, plasma or bronchoalveolar lavage fluid levels of surfactant protein D (SP-D) and soluble receptors for advanced glycation end products (sRAGE)—key markers of epithelial injury—are significantly elevated, accompanying the severe inactivation of endogenous pulmonary surfactants (21). In contrast, systemic inflammatory mediators in non-pulmonary sepsis must reach the lungs via the bloodstream; therefore, the core pathological mechanism of acute lung injury in non-pulmonary sepsis is widespread dysfunction of the vascular endothelial barrier, specifically manifested by severe degradation of the endothelial glycocalyx and a surge in angiopoietin-2 and von Willebrand factor levels (22). This distinct epithelial versus endothelial injury paradigm, determined by the infection’s origin, underscores why the infection focus must be evaluated as a primary covariate when assessing sepsis phenotypes (23). Supporting this concept, a multicenter study on direct versus indirect acute lung injury confirmed that the origin of infection fundamentally shapes the molecular signature of endothelial and epithelial injury, reinforcing the need for source-specific phenotyping (23).

### 2.3 Inflammatory polarization of alveolar macrophages and the local microenvironment

Under physiological homeostasis, resident alveolar macrophages (AMs) are regulated by the surrounding pulmonary epithelial microenvironment, maintaining a low-reactivity phenotype. This prevents excessive immune activation in response to inhaled harmless particles or commensal microbiota. At the molecular level, AMs highly express the CD200 receptor and maintain an anti-inflammatory/pro-inflammatory balance through interactions with CD200-expressing pulmonary epithelial cells (24, 25). Once highly virulent respiratory pathogens (e.g., *Streptococcus pneumoniae* or influenza viruses) breach this defense, pattern recognition receptors (PRRs) in local lung epithelial cells and macrophages are strongly activated, releasing large quantities of chemokines and pro-inflammatory cytokines such as TNF, IL-1β, and IL-6 (26, 27). Peripheral blood monocytes subsequently flood into the alveolar interstitium and differentiate into a new generation of macrophages. Unlike resident AMs, these newly recruited monocyte-derived macrophages lack the tolerogenic imprint of the local microenvironment and retain pro-inflammatory epigenetic programs. They exhibit extreme plasticity and a powerful inflammatory cascade amplification effect, acting as key drivers that propel local infection into systemic sepsis (28).

## 3 Inflammatory phenotypes and endotype characteristics of pulmonary sepsis

### 3.1 Macroscopic inflammatory phenotype

Although phenotypic classification is derived from a heterogeneous critical care population, it exhibits unique distribution patterns and prognostic value within the pulmonary subgroup.

Through latent class analysis (LCA) applied to data from multiple large-scale clinical trials (e.g., HARP-2, FACTT, ALVEOLI, and VALID), two fundamental inflammatory phenotypes have been universally identified: the “hyperinflammatory phenotype” and the “hypoinflammatory phenotype” (29). Across various cohorts, the hyperinflammatory phenotype is consistently associated with higher disease severity (e.g., elevated PRISM III and SOFA scores) and poorer prognoses (29, 30). At the biomarker level, this phenotype features extremely high levels of IL-6, IL-8, soluble tumor necrosis factor receptor-1 (sTNFR1), and severe hypobicarbonemia (30). Conversely, patients with the hypoinflammatory phenotype exhibit compensatory upregulation of markers like MMP-8, lower dependence on vasoactive agents, and improved survival rates (30). The hypoinflammatory phenotype has been further characterized at the proteomic level, revealing distinct patterns of complement and coagulation proteins that differentiate it from the hyperinflammatory subtype and may guide specific biomarker-based enrichment strategies (31). Underlying immune status also dictates phenotype; for example, hematologic malignancies are significantly associated with the hyperinflammatory phenotype (OR = 4.3) (32).

Furthermore, the SENECA cohort study, utilizing machine learning, classified sepsis into four clinical subtypes: α, β, γ, and δ. Among these, the δ (delta) subtype closely aligns with the hyperinflammatory phenotype, characterized by refractory hypotension, hyperlactatemia, and severe hepatic and cardiovascular failure, with persistently high mortality rates. In contrast, the α and β subtypes exhibit milder organ dysfunction and lower levels of systemic inflammation (9).

### 3.2 Microscopic endotypes

While clinical and protein biomarkers capture downstream outcomes, genome-wide transcriptomics provides a molecular-level perspective to elucidate gene regulatory networks and pathogenic mechanisms, defining distinct “endotypes”.

In the UK’s large-scale Genomics in Prospective Cohorts (GAinS) study, leukocyte RNA sequencing of patients with pneumonia and peritonitis-induced sepsis identified two Sepsis Response Signatures (SRS) strongly associated with prognosis:

1. SRS1 (Immunosuppressive Subtype): Patients exhibited classic immune paralysis and exhaustion. Their transcriptomic profile was highly enriched in endotoxin tolerance, apoptosis pathways, T-cell exhaustion, and significant downregulation of human leukocyte antigen (HLA) class II molecules (33). SRS1 is also linked to severe metabolic reprogramming, involving abnormalities in the hypoxia-inducible factor 1α (HIF-1α) and mTOR signaling pathways, which drive immune cells toward inefficient glycolysis (34). Clinically, the 14-day early mortality rate for SRS1 patients reached 22%, significantly higher than the 10% in SRS2 patients (HR 2.4-2.8, *P* < 0.005).
2. SRS2 (Immunocompetent Subtype): Patients maintained relatively intact adaptive immune function and cell proliferation capacity (33). Notably, the high-risk SRS1 endotype was significantly more enriched in the pneumonia cohort than in the peritonitis cohort. By integrating genotyping and RNA-seq data from 638 sepsis patients, this study identified numerous eQTLs and modQTLs, revealing the genetic networks driving these SRS endotypes (35).

Similarly, a multi-cohort RNA-seq meta-analysis classified sepsis into coagulopathic, adaptive, and inflammatory subtypes (36). The coagulopathy subtype carried the highest mortality (30% vs. 16%), while the adaptive subtype contained higher proportions of active CD8+ and memory CD4+ T cells, correlating with better survival. The Dutch MARS cohort study defined four blood gene expression phenotypes (Mars1-4). Mars1, corresponding to severe immunometabolic dysregulation, was consistently associated with 28-day mortality rates as high as 39%-52.8% (37). A Brazilian multicenter study validated these phenotypes in low- and middle-income countries, confirming Mars1’s association with higher mortality (HR = 1.78) (38). Additionally, two transcriptomic phenotypes (USE-1 and USE-2) identified in a Ugandan cohort highlight the global diversity of sepsis endotypes (39).

Crucially, host endotypes are highly dynamic. Sequential transcriptomic sampling revealed that over time, nearly 46% of patients experienced endotype drift; the vast majority evolved from the high-risk SRS1 to the favorable-prognosis SRS2, suggesting the existence of active, dynamic repair mechanisms in underlying gene expression (40, 41). A longitudinal transcriptomic study has now provided direct evidence that sepsis endotypes can shift over time, with nearly half of patients transitioning from the high-risk SRS1 to the SRS2 signature during the first week, emphasizing the importance of repeated sampling for accurate stratification (42).

### 3.3 Are these classification systems interchangeable?

These systems are often cited side by side, as though they identified the same patients, but they were built from different kinds of data — clinical variables in some cases, plasma proteins in others, whole-blood transcriptomes in others — in different cohorts and with different algorithms, and few have been compared with one another in the same patients. **Table 2** compares the principal systems in detail. A patient labelled hypoinflammatory in one system is therefore not necessarily SRS1 in another, and the degree of overlap between systems has never been measured prospectively. Prognosis may also diverge: SRS1 is consistently associated with higher mortality (33), whereas the hypoinflammatory phenotype described in ARDS cohorts has usually carried a lower mortality than the hyperinflammatory phenotype (29, 30). The two labels point in different directions, and they should be reported separately rather than merged (36, 37, 39).

Endotypes also change over time. In a re-analysis of three cohorts, the proportion of patients classified as hyperinflammatory fell from 36.0% on day 0 to 6.3% by day 4 in one cohort, and from 24.8–26.4% to 5.7–13.4% by day 3 in the other two; patients who moved from the hyperinflammatory to the hypoinflammatory phenotype had better 90-day survival than those who remained hyperinflammatory (41). Transcriptomic signatures shift in a similar way, with a substantial proportion of patients changing category during the first week (42). A single baseline measurement is therefore better treated as a working estimate than as a diagnosis, and repeat assessment within 24 to 72 hours is reasonable in patients who deteriorate or fail to improve. Because transcriptomic profiling is not yet routinely available at the bedside, repeated assessment will usually have to rely on simpler protein-based classifiers, such as the three-variable model based on interleukin-8, bicarbonate and soluble tumor necrosis factor receptor-1 (29, 41).

## 4 Pathogen-associated phenotypic characteristics

In hosts with uniform genetic backgrounds, the physical structure, molecular phenotype, and virulence traits of exogenous pathogens—including Gram-negative bacteria, Gram-positive bacteria, viruses, and fungi—activate distinct pathogen-associated molecular patterns (PAMPs). A network-based analysis of pathogen–host interactions in severe pneumonia has revealed that different pathogens engage distinct intracellular signaling modules, which converge on common inflammatory endotypes but diverge in their downstream metabolic consequences, providing a systems-level explanation for pathogen-specific phenotype variation (43). Pathogens are not merely non-specific disease initiators; they are decisive factors that actively reshape the host’s inflammatory phenotype (10). **Table 1** maps the evidence that links each pathogen class to the phenotypes and endotypes discussed below.

### 4.1 Gram-negative bacteria in the lungs

Pathogen characteristics, including species, bacterial load, and primary infection site, have strong independent causal associations with hyperinflammatory and hypoinflammatory phenotypes (10). Gram-negative bacteria possess a profound ability to trigger destructive inflammation. Data show that in patients with pulmonary or bloodstream infections caused by *Escherichia coli*, the median probability of exhibiting a hyperinflammatory phenotype is 0.50. In contrast, for infections caused by *Staphylococcus aureus* (Gram-positive), this probability drops to 0.14 (*P* < 0.001) (10). Single-cell transcriptomics has further confirmed heterogeneity in monocyte responses between Gram-positive and Gram-negative infections (44).

This macroscopic difference stems from microscopic signaling networks: endotoxin activity correlates positively with severe inflammation (ρ = 0.13, *P* = 0.003) (10). Lipopolysaccharide (LPS)—whose core toxic component is lipid A—binds to serum LPS-binding protein and strongly engages the TLR4-MD2-CD14 receptor complex on cell membranes. This leads to explosive downstream activation of the NF-κB transcription factor, triggering massive reactive oxygen/nitrogen species production and a cytokine storm (45).

### 4.2 Gram-positive bacteria in the lungs

Although both fall under Gram-positive infections, pneumococcal-driven pyroptotic epithelial damage and enterococcal-driven low-inflammatory peritoneal infections represent distinct pathophysiological pathways (46). Patients with Gram-positive infections generally exhibit elevated levels of S100A12, globulins, and higher platelet counts, whereas those with Gram-negative infections exhibit more severe systemic inflammation, metabolic suppression, and poorer prognoses.

Extracellular vesicles released by pathogens further amplify these effects. Membrane vesicles released by Gram-positive bacteria, such as Bacillus anthracis and Mycobacterium spp., have immunopathological and immunomodulatory roles similar to those of Gram-negative outer membrane vesicles (47). Pneumolysin released by *Streptococcus pneumoniae* suppresses the initial macrophage pro-inflammatory response (48). Furthermore, they act as sepsis “amplifiers” by releasing large amounts of pro-inflammatory cytokines (IL-1β, IL-18) into the circulation. Thus, for hyperinflammatory phenotypes driven by *Streptococcus pneumoniae*, pyroptosis inhibitors targeting the NLRP3 inflammasome or GSDMD may hold significant therapeutic potential alongside antibiotics (48).

### 4.3 Respiratory viruses

Following the global COVID-19 pandemic, “viral sepsis” has entered the mainstream, accounting for a large proportion of sepsis cases. It uniquely predisposes patients to the dual complications of a hyperinflammatory cytokine storm and profound immunosuppression (49).

Mechanistically, viral pulmonary sepsis radically reshapes host T lymphocyte differentiation (50). The massive release of type I interferon (Type I IFN) triggered by viral infection induces the depletion of lung-resident memory T cells and the abnormal expansion of the Ly6C+ naive CD8+ T cell subset. These develop into functionally abnormal memory CD8+ T cells with distinct transcriptomes and chromatin profiles, resulting in excessive proliferation but flawed tissue localization and reduced cytotoxicity (51).

Additionally, many respiratory viruses exploit host purine metabolism to evade immunity. By expressing CD38 and CD73-like enzymes, they cause a surge in extracellular adenosine, which binds to host low-affinity G protein-coupled receptors. This directly inhibits pro-inflammatory cytokine secretion by effector cells, forcibly weakening host defenses and precipitating a clinical state of “immune paralysis” (52, 53).

### 4.4 Immune failure in fungal pulmonary sepsis

Among patients subjected to prolonged hospitalization, extensive broad-spectrum antibiotics, or with compromised immune systems, the case-fatality rate for sepsis caused by secondary pulmonary fungal infections is extraordinarily high (54, 55). Unlike the acute bacterial inflammatory storm, peripheral blood immune cells in fungal sepsis exhibit a profoundly “exhausted” phenotype characterized by severe immunosuppression (56).

Studies demonstrate that T cells, B cells, and macrophages in this stage are globally dysfunctional. Effector cells show significant downregulation of positive co-stimulatory molecules (e.g., CD28, CD27) and persistently elevated expression of negative immune checkpoint receptors, such as programmed cell death protein 1 (PD-1), its ligand PD-L1, and cytotoxic T-lymphocyte-associated protein 4 (CTLA-4) (57–59). Concurrently, antigen-presenting cells experience overactivation of the Wnt signaling pathway and ROS-induced epigenetic abnormalities, leading to a permanent loss of pathogen clearance capabilities (60). This infection-driven collapse of immune surveillance is tightly linked to the SRS1 transcriptomic endotype.

In clinical practice, bacterial–viral co-infections are common in pulmonary sepsis and have been shown to aggravate the hyperinflammatory response while simultaneously inducing T-cell exhaustion, creating a mixed phenotype that challenges current classification systems (61).

Similarly, polymicrobial pulmonary infections (bacterial–fungal or mixed Gram-negative/positive) are associated with a distinct host response characterized by persistent immune dysregulation and higher 28-day mortality, underscoring the need to incorporate microbiological complexity into future phenotyping models (62).

## 5 Precision treatment strategies based on phenotypes and pathogens

Historically, broad-spectrum antibiotics combined with non-specific organ support formed the cornerstone of pulmonary sepsis management (63). However, as our understanding of phenotypic heterogeneity deepens, it has become evident that uniform interventions ignoring endotypes produce diametrically opposed outcomes in different patient subgroups. Secondary analyses of multiple RCTs have robustly confirmed this paradigm (29, 64) (**Table 1**).

### 5.1 Phenotype dependence of fluid resuscitation strategies

Fluid resuscitation in sepsis remains highly controversial. The FACTT study on severe ARDS identified a critical relationship between phenotype and survival: the effect of fluid strategy on 90-day mortality differed between the two subphenotypes (P = 0.0039 for interaction). In the hypoinflammatory subphenotype, a conservative strategy was associated with lower mortality than a liberal one (18% vs 26%); in the hyperinflammatory subphenotype the pattern was reversed (50% vs 40%) (29). This divergence likely stems from phenotypic differences in vascular endothelial barrier impairment. Specifically, endothelial glycocalyx degradation renders microvessels in the hyperinflammatory state highly sensitive to hydrostatic pressure; aggressive fluid administration directly precipitates fatal pulmonary and organ edema. Direct measurement of glycocalyx shedding products (e.g., syndecan-1 and heparan sulfate) has been shown to predict fluid responsiveness differently across sepsis phenotypes, providing a mechanistic rationale for phenotype-directed fluid management (65).

### 5.2 Controversies surrounding glucocorticoid therapy

The use of glucocorticoids—particularly hydrocortisone—in severe pneumonia and septic shock has been heavily debated. Utilizing a transcriptomic perspective, researchers are untangling this controversy. A secondary analysis of the ADRENAL trial reported no evidence of heterogeneity of treatment effect between the two endotypes overall; however, within the subgroup of patients with pulmonary sepsis, those with the immunoadaptive (IA-P) phenotype had higher mortality with hydrocortisone (OR = 5.55, 95% CrI 1.81-21.2), a finding that requires confirmation (66).

Conversely, an analysis of the VANISH trial reached the opposite conclusion: glucocorticoid therapy was associated with increased mortality in patients with the immunocompetent SRS2 endotype (OR = 7.9, 95% CI 1.6-39.9), while no adverse effects were observed in the immunosuppressed SRS1 group (67).

The root cause of these contradictory findings lies in the nature of glucocorticoids as non-specific, broad-spectrum immunosuppressants. For patients whose immunity is already severely compromised (e.g., SRS1 or Mars1), administering steroids may completely abolish their remaining pathogen clearance capabilities. For patients with relatively intact immune function, whether corticosteroids suppress protective immunity or mitigate immunopathological tissue damage depends primarily on pathogen load and the tissue microenvironment (68).

Notably, the landmark CAPE COD trial recently demonstrated that hydrocortisone significantly reduced 28-day mortality in ICU patients with severe community-acquired pneumonia (6.2% vs. 11.9%, *P* = 0.006), primarily by reducing the need for early organ support, such as mechanical ventilation and vasopressors (67, 69).

### 5.3 Statins and extracorporeal blood purification

Beyond fluids and steroids, other therapies exhibit strong phenotype dependence (70).

Simvastatin, an HMG-CoA reductase inhibitor, improves endothelial function and modulates immunity. In a secondary analysis of HARP-2, patients with the hyperinflammatory subphenotype who received simvastatin had better 28-day survival than those given placebo, while no such difference was seen in the hypoinflammatory subphenotype (71, 72). Because the primary trial was negative overall and used a single dose of simvastatin, this signal still needs confirmation.

Polymyxin B hemoperfusion (PMX-HA) is an extracorporeal blood purification technique that selectively adsorbs circulating endotoxins. In an exploratory *post hoc* analysis of EUPHRATES, patients with septic shock and an endotoxin activity assay between 0.60 and 0.89 who received PMX-HA had lower adjusted 28-day mortality than those who received sham treatment, although the trial was negative overall (73, 74). The analysis was exploratory, and it does not support selecting patients for PMX-HA on the basis of inflammatory phenotype. This underscores that inflammatory markers do not solely represent pathological damage; moderate inflammation is fundamental for resisting pathogen proliferation and initiating tissue repair. Blindly blocking inflammatory cascades is detrimental to patients already in a hyporesponsive state. Furthermore, endotoxemia levels in Gram-negative pulmonary sepsis are often lower than in intra-abdominal infections, necessitating stricter patient selection criteria.

Given the varied therapeutic responses across phenotypes, precision medicine must be tailored. For pulmonary sepsis driven by viral cytokine storms (e.g., severe COVID-19), targeted anti-inflammatory drugs like IL-6 receptor antagonists (e.g., tocilizumab) significantly reduce mechanical ventilation days and mortality (75). For immune-exhausted subtypes (e.g., fungal-driven SRS1), future treatments will shift from anti-inflammatory agents to immunostimulants—such as anti-PD-1/CTLA-4 monoclonal antibodies or IL-7—aimed at reversing T-cell exhaustion and reawakening endogenous host defenses (76). Emerging nanotherapies, such as surface-modified mesoporous silica or biomimetic gold nanomaterials that competitively block LPS-TLR4 binding, also offer novel ways to halt pathogen-induced cascades at their source (77).

A phase 1b, single ascending dose study of an anti-PD-L1 antibody showed that checkpoint inhibition was feasible and well tolerated in the short term, and provided early pharmacodynamic signals (76). It did not show that checkpoint blockade reverses T-cell exhaustion or improves outcomes; both questions still require adequately powered trials with enrichment for the target endotype. The integration of rapid multiplex PCR-based pathogen identification with phenotype-guided algorithms has been proposed to optimize antibiotic de-escalation and immunomodulatory therapy, but its clinical utility in pulmonary sepsis awaits prospective validation (78). Cytokine adsorption using hemoperfusion devices has yielded conflicting results; however, a recent trial suggested that early initiation in patients with a hyperinflammatory phenotype and high IL-6 levels may reduce vasopressor requirements, although mortality benefit remains unproven (79).

### 5.4 Why biomarker-guided trials have not changed practice

Several biomarker-guided immunostimulatory trials have produced pharmacodynamic signals without consistent clinical benefit, and the reasons probably lie more in trial design than in the drugs themselves. Immunostimulation has often been started after the window in which the defect was still reversible. Most trials did not enrich for the abnormality they set out to correct, so only a minority of enrolled patients could have benefited. Many were powered for 28-day mortality, an endpoint that an intervention chiefly reducing organ support is unlikely to move. And enrichment by a single analyte overlooks the fact that immunosuppression and hyperinflammation can coexist in the same patient and change over time. Trials that combine enrichment with repeated phenotyping and pre-specified adaptation rules are therefore more likely to succeed. **Table 3** sets out the therapeutic implications of these phenotypes and the situations in which each intervention should not be used.

## 6 Limitations

This review has several limitations. It is a narrative synthesis, not a systematic review. We searched PubMed/MEDLINE, Web of Science Core Collection, the Cochrane Library, CNKI and Wanfang Data in July and August 2026 using terms derived from the title, and we screened the reference lists of the reviews we retrieved; we did not, however, register a protocol, screen in duplicate, or formally assess risk of bias. The evidence presented here is therefore selective rather than exhaustive.

A considerable part of that evidence comes from populations that overlap with, but are not identical to, pulmonary sepsis, including general sepsis cohorts, ARDS cohorts irrespective of infection source, severe community-acquired pneumonia and COVID-19; such findings should be read as hypothesis-generating for pulmonary sepsis itself. Practical constraints also remain: transcriptomic endotyping is still expensive, laboratory-based and slow relative to the course of critical illness, and phenotypes change over time, so a single measurement can misclassify a patient.

## 7 Conclusions and future prospects

The clinical trajectory of pulmonary sepsis reflects a complex interplay between pathogen-specific factors (species, virulence, and load) and the host immune state (alveolar microenvironment, transcriptomics, and metabolic profile) (**Figure 1**). Over the past decade, clinical phenotypes (e.g., hyper- vs. hypoinflammatory) and transcriptomic endotypes (e.g., SRS1/2, Mars1–4) have significantly improved prognostic risk stratification. Recent insights emphasize that distinct pathogens—whether Enterobacteriaceae driving TLR4-dependent inflammation, viruses inducing Type I IFN-mediated T-cell dysfunction, or Aspergillus triggering PD-1-mediated immunosuppression—shape specific host immune trajectories (10). Consequently, pathogen specificity should be explicitly integrated into current phenotyping frameworks. Moving forward, machine learning models that leverage serial laboratory data offer a feasible strategy for real-time, dynamic phenotyping in the ICU (80).

**Figure 1:**
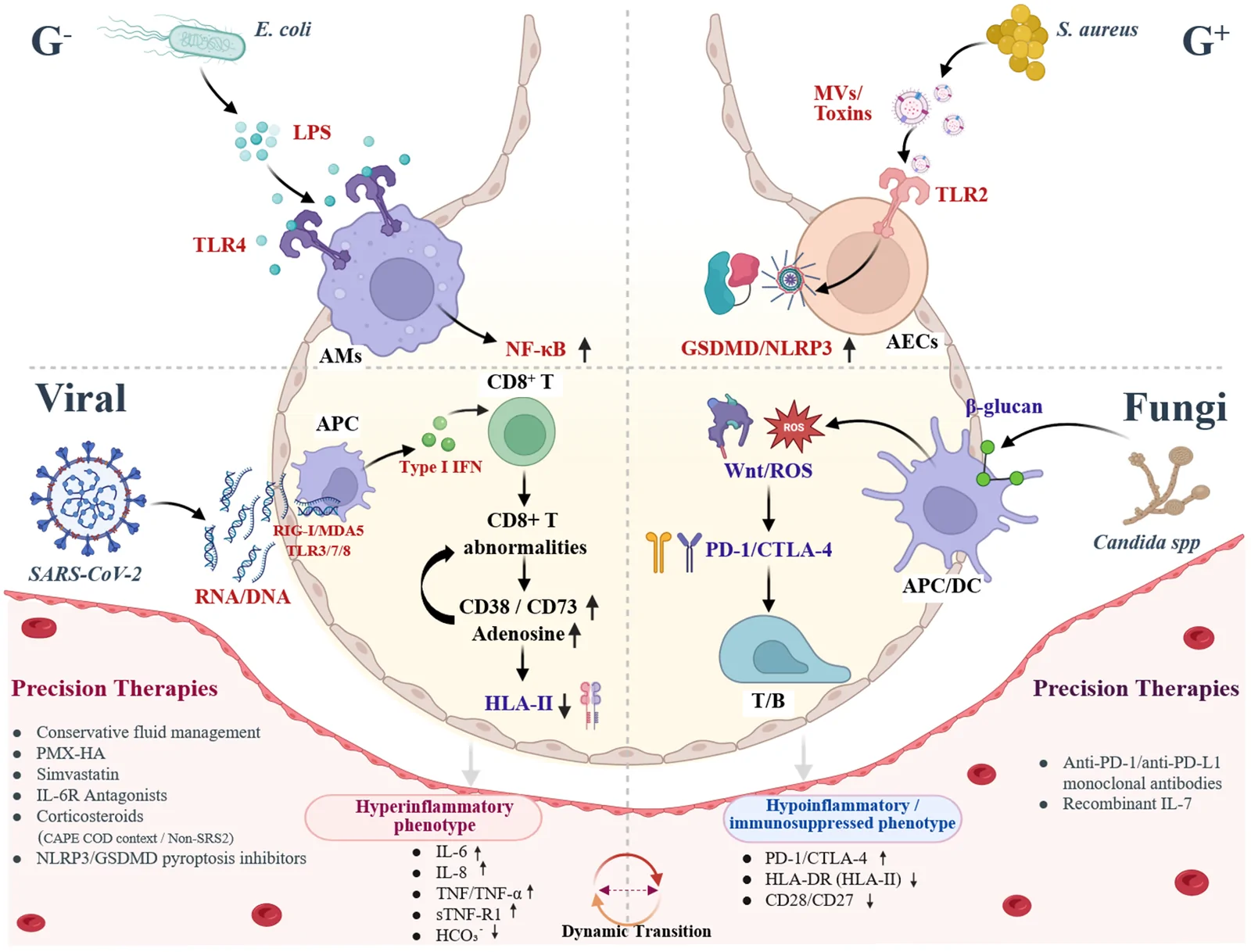
Pathogen-associated host phenotypes and precision therapies in alveolar compartment. In pulmonary sepsis, distinct pathogen classes (Gram-negative/positive bacteria, viruses, fungi) trigger divergent immune cascades within the alveolar compartment via specific PRRs, promoting either explosive inflammation/pyroptosis or profound T-cell exhaustion and APC paralysis. Spillover into the capillary circulation establishes two distinct host-response states — a hyperinflammatory phenotype (IL-6, IL-8, TNF-alpha and sTNFR1 elevated, bicarbonate low) and a hypoinflammatory/immunosuppressed phenotype (PD-1/CTLA-4 elevated, HLA-DR and CD28/CD27 reduced) — with corresponding biomarker signatures. The transcriptomic endotypes SRS1 and SRS2 and the hyper-/hypoinflammatory phenotypes overlap only in part and are not interchangeable, so the correspondence shown here is approximate (Section 3.3). Host endotypes dynamically transition over time, warranting targeted precision therapies—ranging from anti-inflammatory strategies and endotoxin removal to immune-checkpoint blockade and immunostimulants—tailored to specific pathogen profiles and host immune trajectories. AECs, alveolar epithelial cells; AMs, alveolar macrophages; APC, antigen-presenting cell; CTLA-4, cytotoxic T-lymphocyte-associated protein 4; GSDMD, gasdermin D; HLA, human leukocyte antigen; IFN, interferon; IL, interleukin; LPS, lipopolysaccharide; MDA5, melanoma differentiation-associated protein 5; MVs, membrane vesicles; NF-kB, nuclear factor kappa B; NLRP3, NLR family pyrin domain containing 3; PD-1, programmed cell death protein 1; PD-L1, programmed death-ligand 1; PMX-HA, polymyxin B hemoperfusion; RIG-I, retinoic acid-inducible gene I; ROS, reactive oxygen species; SRS1/2, sepsis response signature subtypes 1 and 2; sTNFR1, soluble tumor necrosis factor receptor-1; TLR, Toll-like receptor; TNF-alpha, tumor necrosis factor-alpha.

Translating these phenotyping strategies into clinical precision medicine, however, presents several technical challenges. Rapid point-of-care diagnostics are essential; modalities such as leukocyte biophysical assays and multiplex PCR panels (e.g., BioFire) can now profile pathogen identity and host transcriptomic signatures within hours of admission (81). Furthermore, given the fluid nature of sepsis phenotypes, management cannot rely solely on a baseline evaluation. Next-generation clinical trials will require adaptive designs paired with longitudinal monitoring (82). In this context, circulating cell-free DNA—a marker of tissue damage and neutrophil extracellular trap formation—holds promise for tracking endotype transitions and optimizing the timing of targeted immunomodulations (83). Integrating rapid diagnostics with longitudinal monitoring will be key to capturing dynamic host-pathogen interactions and refining precision therapies for pulmonary sepsis.

Several gaps remain. No prospective trial has yet selected patients with pulmonary sepsis on the basis of an endotype and tested an immunomodulatory strategy against placebo; most of what we know comes from secondary analyses of trials designed for other purposes. The classification systems themselves are only partly comparable, and none has been validated for repeated bedside use. Until those gaps are addressed, the phenotypes and endotypes discussed here are best regarded as tools for stratification and trial design, not as routine clinical tests.

## Glossary

ADRENAL: Adjunctive Corticosteroid Treatment in Critically Ill Patients with Septic Shock
ALVEOLI: Assessment of Low Tidal Volume and Elevated End-Positive End-Expiratory Pressure to Obviate Lung Injury
AMs: Alveolar Macrophages
ARDS: Acute Respiratory Distress Syndrome
CAPE COD: Community-Acquired Pneumonia Evaluation of Corticosteroids in ICU Patients
CTLA-4: Cytotoxic T-Lymphocyte-Associated Protein 4
eQTLs: Expression Quantitative Trait Loci
EUPHRATES: Evaluating the Use of Polymyxin B Hemoperfusion in a Randomized Controlled Trial of Adults Treated for Endotoxemia and Septic Shock
FACTT: Fluid and Catheter Treatment Trial
FORECAST: Focused Outcomes Research in Emergency Care in Acute Respiratory Distress and Sepsis Trials
GAinS: Genomics in Prospective Cohorts/Genomic Assessment of Sepsis
GSDMD: Gasdermin D
HARP-2: Hydroxymethylglutaryl-CoA Reductase Inhibition with Simvastatin in Acute Lung Injury to Reduce Inflammation-2
HIF-1α: Hypoxia-Inducible Factor 1-alpha
HLA: Human Leukocyte Antigen
HMG-CoA: 3-Hydroxy-3-Methylglutaryl-Coenzyme A
HR: Hazard Ratio
ICU: Intensive Care Unit
IFN: Interferon
IL: Interleukin
LCA: Latent Class Analysis
LPS: Lipopolysaccharide
MARS: Molecular Diagnosis and Risk Stratification of Sepsis
Mars1–4: Molecular Diagnosis and Risk Stratification of Sepsis Endotypes 1–4
MMP-8: Matrix Metalloproteinase-8
modQTLs: Modification Quantitative Trait Loci
mTOR: Mammalian Target of Rapamycin
NET: Neutrophil Extracellular Trap
NF-κB: Nuclear Factor Kappa B
NLRP3: NLR Family Pyrin Domain Containing 3
OMVs: Outer Membrane Vesicles
OR: Odds Ratio
PAMPs: Pathogen-Associated Molecular Patterns
PCR: Polymerase Chain Reaction
PD-1: Programmed Cell Death Protein 1
PD-L1: Programmed Death-Ligand 1
PMX-HA: Polymyxin B Hemoperfusion
PRR: Pattern Recognition Receptor
PRISM III: Pediatric Risk of Mortality III
RCT: Randomized Controlled Trial
RNA/RNA-seq: Ribonucleic Acid/RNA Sequencing
ROS: Reactive Oxygen Species
S100A12: S100 Calcium Binding Protein A12
SENECA: Sepsis Endophenotyping in Emergency Care
SOFA: Sequential Organ Failure Assessment
SP-D: Surfactant Protein D
sRAGE: Soluble Receptor for Advanced Glycation End Products
SRS/SRS1/SRS2: Sepsis Response Signature/Subtypes 1 & 2
sTNFR1: Soluble Tumor Necrosis Factor Receptor-1
TNF/TNF-α: Tumor Necrosis Factor/Tumor Necrosis Factor-alpha
USE-1/USE-2: Ugandan Sepsis Endotypes 1 & 2
VALID: Validation of Acute Lung Injury Diagnostics
VANISH: Vasopressin vs Norepinephrine in Patients with Septic Shock
